# The molecular evolution of four anti-malarial immune genes in the *Anopheles gambiae *species complex

**DOI:** 10.1186/1471-2148-8-79

**Published:** 2008-03-06

**Authors:** Aristeidis Parmakelis, Michel A Slotman, Jonathon C Marshall, Parfait H Awono-Ambene, Christophe Antonio-Nkondjio, Frederic Simard, Adalgisa Caccone, Jeffrey R Powell

**Affiliations:** 1 Department of Ecology and Evolutionary Biology, Yale University, 21 Sachem Street, 06511, New Haven, CT, USA; 2 Organisation de Coordination pour la Lutte Contre les Endémies en Afrique Centrale (OCEAC), P.O. Box 288, Yaoundé, Cameroon; 3 Institute de Recherche pour le Développement (IRD), UR016, BP 1857, Yaoundé, Cameroon; 4 Department of Biology, University of Crete, P.O. Box 2208, GR-71409, Heraklion, Crete, Greece; 5 Department of Biology, Southern Utah University, Science Center 105, 84720, Cedar City, UT, USA

## Abstract

**Background:**

If the insect innate immune system is to be used as a potential blocking step in transmission of malaria, then it will require targeting one or a few genes with highest relevance and ease of manipulation. The problem is to identify and manipulate those of most importance to malaria infection without the risk of decreasing the mosquito's ability to stave off infections by microbes in general. Molecular evolution methodologies and concepts can help identify such genes. Within the setting of a comparative molecular population genetic and phylogenetic framework, involving six species of the *Anopheles gambiae *complex, we investigated whether a set of four pre-selected immunity genes (*gambicin*, *NOS*, *Rel2 *and *FBN9*) might have evolved under selection pressure imposed by the malaria parasite.

**Results:**

We document varying levels of polymorphism within and divergence between the species, in all four genes. Introgression and the sharing of ancestral polymorphisms, two processes that have been documented in the past, were verified in this study in all four studied genes. These processes appear to affect each gene in different ways and to different degrees. However, there is no evidence of positive selection acting on these genes.

**Conclusion:**

Considering the results presented here in concert with previous studies, genes that interact directly with the *Plasmodium *parasite, and play little or no role in defense against other microbes, are probably the most likely candidates for a specific adaptive response against *P. falciparum*. Furthermore, since it is hard to establish direct evidence linking the adaptation of any candidate gene to *P. falciparum *infection, a comparative framework allowing at least an indirect link should be provided. Such a framework could be achieved, if a similar approach like the one involved here, was applied to all other anopheline complexes that transmit *P. falciparum *malaria.

## Background

Vector-borne diseases such as malaria and dengue constitute a major obstacle to socio-economic development in much of the tropics and remain high on the list of priorities for the improvement of public health. Unlike other infectious diseases, vector-borne diseases stand out because of their complex mode of transmission, requiring the transition from man to man or animal to man through an arthropod vector. This method of transmission implies the simple principle that removal of the vector will lead to the elimination of the disease [1]. This principle has been verified historically, since whenever control of an insect-borne disease has been achieved, this has most often been done through the control of the vector rather than the direct control of the disease through drugs or vaccines. The only exception has been yellow fever, for which a functional vaccine was developed early on [2]. Having this historical fact in mind, research has focused on controlling the vector, and this resulted in an increased output of medical entomological research over the last ten years or so. The best example of this is the acquisition of the complete genome sequence of *Anopheles gambiae *[3], the major malaria vector in sub-Saharan Africa and of *Aedes aegypti *[4], the major vector of yellow fever and dengue.

The wealth of results obtained has now led medical entomologists towards the development of novel ideas that take full account of this new knowledge. One such idea in which much research is invested is the "construction" of "new mosquitoes" that would be unable to transmit malaria or other diseases [5,6]. This "new mosquito" would be based on transgenic strains carrying genes that make them refractory to their parasites. After almost two decades in effort, the technology for creating transgenic mosquitoes has been developed and encouraging results have sprouted from this research [7-9]. However, there is still the need to identify those refractory genes best suited for "constructing" transgenic mosquitoes.

In the case of malaria, the insect's immunity genes have been considered as very good candidates, since following the sequencing of the *An. gambiae *genome [3] a large set of genes that mediate mosquito susceptibility to *Plasmodium *infections has been identified [10-16]. However, if the insect innate immune system is to be used as a potential blocking step in transmission of malaria, then it will require targeting one or a few genes of highest relevance and ease of manipulation. The problem, then, is to identify those of highest importance to malaria infection. By and large, the immune responses are adaptive for the mosquito. Therefore, modification of such genes runs the risk of decreasing the mosquito's ability to stave off infections by microbes in general, thereby decreasing mosquito fitness and lessening the chance to become established in the natural population. Ideally, one would want to identify a gene, or part of a gene, that specifically targets the particular pathogen of interest. Furthermore, it is crucial that the gene(s) used in any attempt to block transmission in natural populations focus on genes specific to the relevant mosquito and *Plasmodium *species, since it has been suggested that the defense exhibited by the vector species, varies depending on the invading *Plasmodium *species [11].

In this study we focus on the species of the *An. gambiae *complex and we assess the genetic polymorphism of four genes (*gambicin*, *NOS*, *Rel2 *and *FBN9*) that have been identified as part of the innate immune system of *An*.*gambiae*. The *An. gambiae *complex is composed of seven closely related species, i.e. *An. gambiae*, *Anopheles arabiensis*, *Anopheles melas, Anopheles merus*, *Anopheles bwambae *and *Anopheles quadriannulatus *A and B. The first two species are the major vectors of human malaria in sub-Saharan Africa, with *An. melas *and *An. merus *being intermediate in importance. The last three species are highly zoophilic and are never or rarely exposed to the human *Plasmodium falciparum*.

The studied loci have been implicated in the resistance towards *Plasmodium *infections largely through microarray and RNAi experiments [11,17-19]. Molecular population genetics and phylogenetics is an independent approach that could verify that evolutionary patterns re-enforce implications from laboratory studies. Within a comparative framework, we evaluate the possibility that these four genes evolve, in the lineages of the major malaria vectors, under selection imposed by the direct or indirect interaction with the *Plasmodium*. The approach relies on the prediction that if *Plasmodium *infection affects the mosquito's fitness, we may expect the accumulation of adaptive amino acid substitutions in those anti-malarial genes that are crucial in specifically limiting *Plasmodium *infection in vector species, whereas such changes are less likely to be found in closely related species that have historically limited interaction with the parasite. An assumption of this approach is that the *Anopheles *species have exhibited an adaptive response to *P*.*falciparum *infection which is suggested by several lines of evidence [20].

## Results

### Polymorphism, divergence and McDonald-Kreitman tests

#### Gambicin

A fragment of 589 to 682 bp (Table 1) was amplified from all specimens. This fragment includes all the coding sequence of the gene, consisting of 246 bp. A total of 59 sequences (Table 2) including the whole coding region of this gene were obtained from six species of the *An. gambiae *complex. Out of the 59 sequences 37 represented different alleles. The nucleotide diversity (Pi) varied from 0.000 to 0.024 and 0.001 to 0.007 in the synonymous and non-synonymous sites, respectively (Table 2). There were very few mutations (4 mutations in 3 pairwise comparisons) shared between species, whereas there were four alleles out of the 37, that were shared between them. For the coding region, Dxy (average number of nucleotide substitutions per site between species) ranged from 0.006 to 0.014. Very few fixed differences were present between species, and in most comparisons no fixed non-synonymous differences were found (Table 3). Not surprisingly therefore, the McDonald-Kreitman tests did not indicate positive selection.

**Table 1:** Sequences of primers used in the study for the amplification of the four anti-malarial immunity genes. If PCR_1 _was not succesfull or produced very low signal a nested PCR was applied.

	*PCR_1_*	*PCR_nested_*
*Gambicin*	Gamb_exon_313_1505F	Gamb_exon_46_692F
	TGAATCCCCTCGGCTCGCTG	CTGAACGCCGTCACAAGTGC
	Gamb_exon_313_1505R	Gamb_exon_46_692R
	TGCAGTGAGTTATGTCACAAGC	TGGCACTGATTAAACCGCTTG

*NOS*	*NOS*_exon_30528F	*NOS*_exon_30705F
	GTGGAYGGAATYATYGAGCG	GGTGTCTACAAATCKGGGA
	*NOS*_exon_31858R	*NOS*_exon_31692R
	MCGCSYTACTTACCCGCAGCG	CGAKTCCGCCTCYTTGAGGGC

*Rel2*	Rel2_exon_415F	Rel2_exon_504F
	ACACCGTCCTGTCGATGGAC	GGTCGCACCTATGCCAGTGC
	Rel2Frag1Rev	Rel2_exon_1275R
	GATGCCCATACCCTGGAAGG	ACACCCTCCGATGGTTCAGC

*FBN9*	*FBN9*_217F	*FBN9*_264F
	TCCGACCTCCACCGGGTACG	ACTACCTACAGTACAAGCTGCTC
	*FBN9*_1149R	*FBN9*_1075R
	AGCCATGCCCTGGTGCGAGC	GGCAGTTGTTGTACCACCAG

**Table 2:** Sequence data and polymorphism parameters of the four amplified immunity genes in the species of the *An. gambiae *complex. Length of sequences, exons and number of exons refer to the fragments sequenced in this study. Alleles were inferred based on the coding regions of the sequences.

	Number of individuals	Length of sequences obtained (bp)	Number of exons/length of each exon sequence (bp) analysed	Number of sequences obtained per species (number of alleles per species)	Polymorphic sites		Nucleotide diversity (Pi)	
					
					Syn.	Non-Syn.	Syn.	Non-Syn.
*Gambicin*								

***An. arabiensis***	6	646–677	3/75, 90, 81	10 (8)	5	5	0.002	0.007
***An. bwambae***	6	667–675	3/75, 90, 81	11 (7)	4	5	0.020	0.006
***An. gambiae***	5	666–672	3/75, 90, 81	9 (8)	4	4	0.024	0.006
***An. melas***	6	589–672	3/75, 90, 81	11 (4)	0	5	0.000	0.004
***An. merus***	5	664–682	3/75, 90, 81	8 (4)	2	1	0.008	0.001
***An. quadriannulatus***	5	663–682	3/75, 90, 81	10 (6)	5	4	0.019	0.004

**Total**	33			59 (37)				

*NOS*								

***An. arabiensis***	5	997–1006	3/201, 432, 138	5 (5)	19	2	0.050	0.001
***An. bwambae***	7	997–1008	3/201, 432, 138	11 (8)	10	7	0.021	0.003
***An. gambiae***	6	1006–1007	3/201, 432, 138	7 (7)	16	6	0.029	0.003
***An. melas***	5	1004–1007	3/201, 432, 138	8 (5)	3	4	0.007	0.002
***An. merus***	6	993–1001	3/201, 432, 138	10 (8)	15	4	0.028	0.001
***An. quadriannulatus***	5	1002–1006	3/201, 432, 138	5 (5)	7	2	0.019	0.001

**Total**	34			46 (38)				

*Rel2*								

***An. arabiensis***	10	780–785	2/372, 339	20 (18)	15	10	0.031	0.004
***An. bwambae***	6	712–778	2/372, 339	6 (1)	0	1	0.000	0.000
***An. gambiae***	14	732–794	2/372, 339	16 (11)	19	6	0.020	0.004
***An. melas***	8	761–800	2/372, 339	10 (5)	3	2	0.008	0.001
***An. merus***	7	665–796	2/372, 339	11 (6)	2	4	0.003	0.002
***An. quadriannulatus***	8	762–803	2/372, 339	11 (8)	9	4	0.013	0.002

**Total**	53			74 (49)				

*FBN9*								

***An. arabiensis***	7	789–807	1/807	12 (12)	40	7	0.065	0.003
***An. bwambae***	7	787–807	1/807	8 (3)	18	2	0.040	0.001
***An. gambiae***	7	764–807	1/807	12 (12)	41	1	0.079	0.000
***An. melas***	7	776–791	1/791	7 (7)	9	0	0.021	0.000
***An. merus***	7	788–807	1/807	11 (10)	10	4	0.016	0.002
***An. quadriannulatus***	7	780–807	1/807	10 (10)	18	6	0.034	0.002

**Total**	42			60 (54)				

**Table 3:** MacDonald-Kreitman tests on *gambicin *and *NOS*.

	*Gambicin*					*NOS*				
	Fixed		Polymorp.			Fixed		Polymorp.		

	S	NS	S	NS	p-value	S	NS	S	NS	p-value

*gam-ara*	0	0	9	8	--	1	0	33	8	n.s.
*gam-qua*	0	0	9	8	--	2	0	22	8	n.s.
*gam-mel*	1	0	4	9	n.s.	2	0	17	9	n.s.
*gam-mer*	0	0	6	5	--	2	0	29	10	n.s.
*gam-bwa*	0	0	8	9	--	1	0	24	13	n.s.
*ara-qua*	0	0	9	8	--	2	1	25	4	n.s.
*ara-mel*	0	0	5	10	--	3	1	22	5	n.s.
*ara-mer*	0	0	7	5	--	1	1	31	6	n.s.
*ara-bwa*	0	0	9	10	--	0	1	29	9	n.s.
*qua-mel*	0	0	5	9	--	3	0	10	6	n.s.
*qua-mer*	0	0	7	5	--	1	0	22	6	n.s.
*qua-bwa*	0	0	9	9	--	1	0	15	9	n.s.
*mel-mer*	1	0	2	6	n.s.	3	0	17	7	n.s.
*mel-bwa*	1	0	4	10	n.s.	2	0	13	10	n.s.
*mer-bwa*	0	0	6	6	--	0	0	23	11	--

Species names are abbreviated as follows: *An. arabiensis*: *ara*, *An. bwambae*: *bwa*, *An. gambiae*: *gam*, *An. melas*: *mel*, *An. merus*: *mer*, *An. quadriannulatus*: *qua*. S: synonymous mutations, NS: non-synonymous mutations, n.s.: non significant.

#### NOS

A total of 46 sequences were produced from the studied species (Table 2). The sequences produced varied from 993 to 1007 bp, of which 771 bp were coding. Out of the 46 sequences 38 were different alleles. Although there were some shared polymorphisms (10 in six pairwise comparisons) between the species, there were no alleles shared between them. Dxy ranged from 0.009 to 0.017 for the coding region. Nucleotide diversity (Pi) ranged between 0.007 to 0.050 and 0.001 to 0.003 in the synonymous and non-synonymous sites, respectively. A single fixed replacement substitution was observed in all pairwise comparisons (Table 3) with *An. arabiensis *(except with *An. gambiae*). However, none of the McDonald-Kreitman tests were significant.

#### Rel2

We obtained 74 sequences from six species. The fragments amplified in all specimens ranged from 665 to 803 bp. Of these approximately 710 bp were coding sequence (Table 2). Out of the 74 sequences 49 were different alleles (Table 2). There were a few polymorphisms shared between species (nine in two pairwise comparisons) but there were no shared alleles. Dxy ranged from 0.014 to 0.037 between species. The nucleotide diversity (Pi) in the synonymous as well as in the non-synonymous sites was quite low varying from 0.000 to 0.031 in the synonymous sites, and from 0.000 to 0.004 in the non-synonymous sites. As was the case in the previous genes, the McDonald-Kreitman tests of positive selection did not show a significant excess of fixed non-synonymous differences between the species, although there were fixed replacement substitutions present in all the pairwise species comparisons (Table 4).

**Table 4:** MacDonald-Kreitman tests on *Rel2 *and *FBN9*.

	*Rel2*					*FBN9*				
	Fixed		Polymorp.			Fixed		Polymorp.		

	S	NS	S	NS	p-value	S	NS	S	NS	p-value

*gam-ara*	1	0	26	13	n.s.	0	0	63	7	--
*gam-qua*	2	1	24	9	n.s.	3	0	57	7	n.s.
*gam-mel*	1	2	22	8	n.s.	5	0	48	1	n.s.
*gam-mer*	1	1	18	8	n.s.	3	0	49	5	n.s.
*gam-bwa*	2	2	18	6	n.s.	2	0	52	2	n.s.
*ara-qua*	2	1	20	12	n.s.	2	0	55	13	n.s.
*ara-mel*	7	8	17	11	n.s.	6	0	49	7	n.s.
*ara-mer*	2	1	16	12	n.s.	3	0	49	11	n.s.
*ara-bwa*	5	2	15	10	n.s.	4	0	51	8	n.s.
*qua-mel*	6	7	11	6	n.s.	9	0	26	6	n.s.
*qua-mer*	4	3	10	7	n.s.	4	0	26	10	n.s.
*qua-bwa*	4	3	8	5	n.s.	7	0	34	8	n.s.
*mel-mer*	3	1	5	6	n.s.	13	0	19	4	n.s.
*mel-bwa*	11	6	3	3	n.s.	5	0	26	2	n.s.
*mer-bwa*	9	2	2	4	n.s.	5	0	28	6	n.s.

Species names are abbreviated as in Table 3. S: synonymous mutations, NS: non-synonymous mutations, n.s.: non significant.

#### FBN9

We successfully determined 60 sequences from the six species all together. The fragments amplified in all specimens ranged from 764 to 807 bp, and they were all coding sequence (Table 2). Of these 60 sequences, 54 were different alleles (Table 2). There were a lot of mutations (48 mutations in 10 pairwise comparisons) shared between species, but again there were no alleles shared. Dxy ranged from 0.016 to 0.029 between species. Nucleotide diversity (Pi) in the synonymous sites was higher compared to the previous genes and varied from 0.016 to 0.079. However, it was quite low in the non-synonymous sites since it ranged from 0.000 up to 0.003. As presented in Table 4 there is not even a single fixed non-synonymous substitution between the species of the complex. Consequently, the McDonald-Kreitman tests were negative regarding positive selection acting on the gene (Table 4).

**Figure 1:**
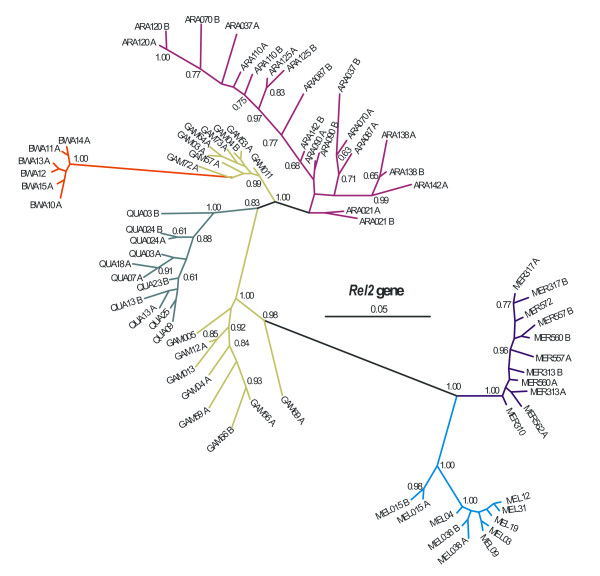
***Rel2 *Bayesian Inference Tree**. 50% majority-rule consensus Bayesian (unrooted) tree of *Rel2*. Numbers on branches are the posterior probabilities of clades, only values above 0.5 are presented. Species names have been abbreviated as follows: ARA: *An. arabiensis*, BWA: *An. bwambae*, GAM: *An. gambiae*, MEL: *An. melas*, MER: *An. merus*, and QUA: *An. quadriannulatus*. The number following the species abbreviation refers to the individual specimen code, whereas the letters A and B differentiate between the two alleles of a single individual specimen. Details of the Bayesian analysis can be provided upon request.

A second series of McDonald-Kreitman tests were performed for all the genes, but this time the alleles were not grouped according to species. They were grouped according to their phylogenetic relationships, as indicated by the inferred phylogenetic trees. For example in *Rel2*, a set of the *An*. *gambiae *alleles were pooled together with the *An. bwambae *sequences to form a single group, as indicated by the phylogenetic tree of figure 1. This group of alleles was subsequently contrasted to the alleles of the remaining species. Again no signs of positive selection could be detected.

### Phylogeny and maximum likelihood tests for selection

**Figure 2:**
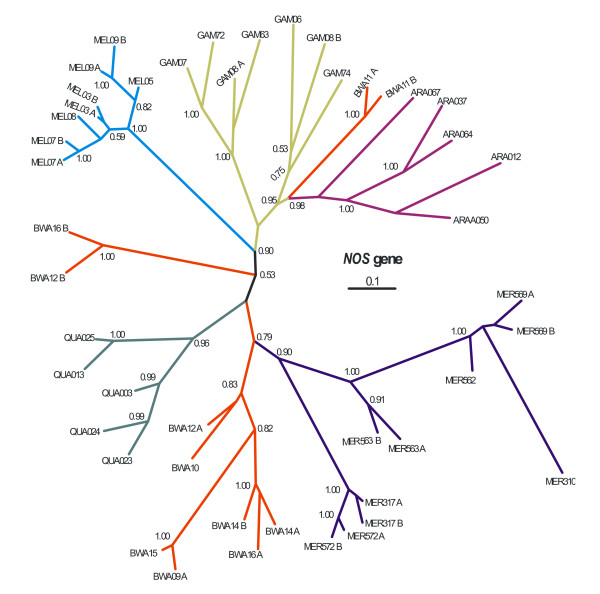
***NOS *Bayesian Inference Tree**. 50% majority-rule consensus Bayesian (unrooted) tree of *NOS*. Numbers on branches are the posterior probabilities of clades, only values above 0.5 are presented. Species names have been abbreviated as follows: ARA: *An. arabiensis*, BWA: *An. bwambae*, GAM: *An. gambiae*, MEL: *An. melas*, MER: *An. merus*, and QUA: *An. quadriannulatus*. The number following the species abbreviation refers to the individual specimen code, whereas the letters A and B differentiate between the two alleles of a single individual specimen. Details of the Bayesian analysis can be provided upon request.

Since no non-synonymous fixed differences were found in any species pairwise comparisons for *Gambicin *and *FBN9 *(Table 3, 4) only *NOS *and *Rel2 *were subjected to the PAML analysis. The phylogenetic trees for these two genes, produced from the bayesian analysis, and on which the various models of the PAML software were evaluated for their goodness of fit, are presented in figures 1 and 2.

In the *NOS *tree (Figure 2) only *An. merus*, *An. melas *and *An. quadriannulatus *form strongly supported monophyletic groups. Some alleles of *An*.*bwambae *(BWA16 B/BWA12 B and BWA11 A/BWA11 B) are placed very far apart from their conspecific sequences. Furthermore, each one of these two sets of *An. bwambae *alleles, seems to be closely related to a different *Anopheles *species. The alleles BWA16 B/BWA12 B are placed within the clade hosting *An. merus*, *An. bwambae *and *An. quadriannulatus*, whereas the alleles BWA11 A/BWA11 B seem to be more closely related to *An. gambiae *and *An. arabiensis*. At the same time, the alleles of *An. gambiae *seem to be forming two separate clades that are firmly to each other and are ambiguously related to the *An. arabiensis *clade.

In the *Rel2 *tree (Figure 1), the alleles of most species form strongly supported monophyletic groups and the relationships between the species are well resolved (posterior probabilities above 0.80). As was the case in *NOS*, the alleles of *An. gambiae *form two separate clades that are placed apart. In the case of *Rel2*, one of the *An. gambiae *groups is closely related to *An. bwambae*, and the other is part of a broader clade that incorporates both the *An. merus *and the *An. melas *clades.

In the case of the *NOS *gene, only the likelihood ratio test of M0 versus M3 (Table 5) was statistically significant and in favor of variable selection pressure among sites. All other likelihood ratio tests comparing the among sites models, were not in favor of an ω value greater than one among sites (Table 5). Similar results were obtained for *Rel2*, where again only the comparison between models M0 and M3 (Table 5) indicated variable selection pressure acting on the amplified *Rel2 *fragment.

**Table 5:** Likelihood ratio tests in *NOS *and *Rel2 *between models that allow codon sites to evolve under positive selection (M3, M2a, M8) and those that do not (M0, M1a, M7).

AMONG SITES TESTS							
***NOS***				***Rel2***			

Model	*Ln*	*2ΔLn^a^*	*p-value*	*Ln*	*2ΔLn^a^*	*p-value*	*df*

M0	-1981.64533	12.83610	0.0121*	-1384.06495	15.63108	0.0036*	4
M3	-1975.22728			-1376.24941			
							
M1a	-1975.76521	1.04519	n.s.	-1376.33264	0.00004	n.s.	2
M2a	-1975.24262			-1376.33262			
							
M7	-1975.59590	0.72744	n.s	-1376.64901	0.77912	n.s.	2
M8	-1975.23218			-1376.25945			

* Significant *p-value *at 0.05 significance level; *df*: degrees of freedom

^a ^This quantity is compared to the critical values of a chi-square distribution with the respective degrees of freedom

Regarding the branch-site tests for the *NOS *and *Rel2 *(Table 6), regardless of whether *An. gambiae *or *An. arabiensis *were designated as the foreground branches, the likelihood ratio tests were not in favor of positive selection acting on at least some codons of the foreground branches in either gene. The branch-site test 2 for *NOS*, was also applied to a phylogenetically designated "*gambiae*" clade. By this we mean that we considered as the *gambiae *clade (foreground branch) not only the alleles of *An*. *gambiae*, but those of *An. arabiensis *and *An. bwambae *that are ambiguously related to the *An. gambiae *alleles, as well (Figure 2). The result of the branch-site test 2 was negative in this case as well.

**Table 6:** Branch-site test 2 in *NOS *and *Rel2 *with *An. arabiensis *and *An. gambiae *designated as the foreground branches.

Branch-site test 2				
***NOS***				

	*Ln*	*2ΔLn*	*p-value*	*df*

*An. arabiensis: *foreground branch				
model 2, ω = 1	-1780.287415	0.61390	n.s.	1
model 2, ω free to vary	-1779.980464			
				
*An. gambiae: *foreground branch				
model 2, ω = 1	-1779.79876	0.00000	n.s.	1
model 2, ω free to vary	-1779.79876			

***Rel2***				

	*Ln*	*2ΔLn*	*p-value*	*df*

*An. arabiensis: *foreground branch				
model 2, ω = 1	-1376.33322	0.00100	n.s.	1
model 2, ω free to vary	-1376.33272			
				
*An. gambiae: *foreground branch				
model 2, ω = 1	-1374.86019	0.41907	n.s.	1
model 2, ω free to vary	-1374.65065			

*df*: degrees of freedom

## Discussion

The nucleotide diversity (Pi) of all studied loci was quite low in all the species of the *An. gambiae *complex both in the synonymous and the non-synonymous substitutions (Table 2). *Gambicin *exhibited the lowest levels of variation and *FBN9 *the least (Table 2). The levels of divergence of these loci between the different species were low as well. Among the four analyzed loci, *Rel2 *and *FBN9 *were the most divergent, with Dxy values range from 1.4 to 3.7% and 1.6 to 2.9%, respectively.

In the *gambicin *locus it can be seen that in some species pairwise comparisons the fixed differences (synonymous and non-synonymous) are zero (Table 3). One could argue that this could be the result of introgression between the species and/or the continued segregation of inherited ancestral polymorphisms. However, we argue that these two processes have not equally affected the observed pattern. Introgression cannot be ruled out between the lineages of *An*. *gambiae *and *An. arabiensis*, since in the phylogenetic tree of the respective gene (see Additional file 1), the alleles of *An. arabiensis *cluster together with the *An*. *gambiae *alleles. Evidence for introgression between *An. gambiae *and *An. arabiensis *has been reported previously [20-23]. At the same time, the role of the shared ancestral polymorphisms in the observed pattern is limited since in *gambicin *the shared polymorphisms between the species were in the majority of the pairwise comparisons zero (see results). We estimated (results not shown) the level of divergence between species using the non-coding regions of the *gambicin *gene. The net between species average Kimura two-parameter distance [24] as estimated by MEGA v.3.1 [25], ranged between 0.4% (*An. gambiae *versus *An. arabiensis*) and 3.8% (*An. melas *versus *An. arabiensis*). This level of divergence is quite low compared to the respective level of divergence for *Rel2 *and *NOS *(see below). The complete absence of fixed non-synonymous substitutions between pairwise species comparisons in the *gambicin *locus indicates that purifying selection is the major force that shapes the observed evolutionary pattern of *gambicin *(Table 3). Gambicin has been found to be an important antibacterial peptide, that is however, marginally lethal to *P. berghei *[19] and has no specificity towards *P. falciparum *[11]. More likely, this gene as many others belonging to the antimicrobial defense system of the mosquito, evolve under the selection constrains imposed by the bacteria that the mosquitoes encounter during their lifetime. As Dong et al. (2006) also conclude there is little reason to believe that *gambicin *has undergone major adaptations to malaria.

In the *NOS *locus, a great number of polymorphic sites were recorded in all species pairwise comparisons (Table 3). However, the fixed non-synonymous difference was one in each species comparison of *An. arabiensis *with the other species (except *An. gambiae*). As was the case with the *gambicin *locus, in the phylogenetic tree of *NOS*, signs of introgression between *An. gambiae *and *An. arabiensis *are evident (Figure 2). Moreover, two alleles originating from *An. bwambae*, were placed within the *An. gambiae*/*An. arabiensis *clade, another two alleles were placed very close to *An. melas *and the majority of the alleles formed a separate and relatively well supported monophyletic clade. The case for genetic introgression between *An. bwambae *and *An. gambiae *has been made previously [26], and this is reflected here as well. However, we consider that in the case of *NOS*, we are also witnessing sharing of ancestral polymorphisms between the species pairs *An. bwambae*/*An. merus *and *An. bwambae*/*An. quadriannulatus*. We claim this since out of ten shared polymorphisms, four were shared between the previously mentioned pairs of species.

The expression pattern of *NOS *was found to vary depending on the *Plasmodium *species infecting *An. gambiae *[18], implying that the differential expression pattern is the effect of co-evolution between the host and its specific parasite. The present study does not add support to this interpretation since both the McDonald-Kreitman tests and the PAML analysis did not detect any signs of positive selection acting on the *NOS *gene. Even though the PAML analysis indicated that varying selective pressure is acting on the codons of the *NOS *fragment amplified (Table 5), all other likelihood models that allowed for positive selection to be acting on some codons or branches of the *NOS *fragment, produced negative results (Table 5, Table 6).

In the phylogenetic tree of *Rel2 *(Figure 1) there is a group of *An. gambiae *alleles that are very closely related to alleles of *An. bwambae*. Shared polymorphisms in the *Rel2 *locus are detected between *An. arabiensis *and *An. gambiae *but not between *An. gambiae *and *An. bwambae*. Consequently, the unexpected clustering of the *An. gambiae *alleles with *An. bwambae*, is more likely due to introgression between the two species, as noted above for other genes.

At the *Rel2 *locus the level of nucleotide differences among species (Table 4) were comparable to those observed in the *NOS *locus. However, at the *Rel2 *locus, there are several fixed non-synonymous differences between certain pair of species. For example, between *An. arabiensis *and *An. melas *there were eight non-synonymous fixed differences. Neither the McDonald-Kreitman nor the maximum likelihood tests for selection provide evidence of positive selection in this gene. *Rel2*, like *gambicin *and *NOS *was found to be under purifying selection. As was the case in *NOS*, the maximum likelihood analysis of PAML, pointed to a varying selective pressure among sites (M0 vs M3, p-value = 0.0036: Table 5), but no signs of positive selection were detected in the site by site or the branch-site analysis (Table 5, Table 6).

*Rel2 *regulates the expression of the antibacterial genes *CEC1*, *GAM1*, *DEF1*, *CEC3*, and key malaria parasite antagonists, *LRIM1*, *TEP1*, and *TEP4 *in *An. gambiae *[17]. Thus, *Rel2 *is regulating the expression pattern of many and diverse genes of the *Anopheles *innate immune system, among which at least two (*LRIM1 *and *TEP1*) have very strong anti-malaria activity. However, the remaining genes regulated by *Rel2 *are, as far as known, antibacterial peptides. Therefore, even though *Rel2 *may be involved in the defense against malaria infection, its involvement in the expression pattern of many antimicrobial genes may greatly reduce the possibility of this gene specifically exhibiting an evolutionary response to *Plasmodium*. However the comparative approach applied here did detect purifying selection acting on the amplified *Rel2 *fragment from the six species of the *An. gambiae*, as would be expected in a locus coding for a product that serves as the regulator of the expression of the general antimicrobial defense of the organism. Because *Rel2 *is mostly involved in antibacterial defense, why are we not witnessing positive selection imposed by the bacterial pathogens? This type of positive selection has been reported for *GNBP1*, *GNBP2 *and *Relish *in termites and was related to the diverse microbes encountered in different habitats [27]. In the present study, there are significant ecological differences among the studied species. For example the species *An. melas *and *An. merus *favour brackish water for immature development, in contrast to the freshwater species *An. gambiae *and *An. arabiensis*. Even though ecological differences among the studied species are evident, no positive selection could be detected. At this point we have to point that the *Rel2 *fragment targeted in our study resides in the N-terminal homology domain region of Rel2, whereas positive selection has been detected [27] in the spacer connecting this region to the C-terminal ankyrin repeat region (see 27).

The *FBN9 *locus is characterized by a very large number of polymorphic site differences (mainly synonymous) between the species (Table 4). In the phylogenetic tree of *FBN9 *(see Additional file 2) the alleles of *An. gambiae *are interspersed across the tree, whereas the alleles of the remaining species form strongly supported monophyletic groups ambiguously connected to each other. Once more the observed pattern could be the result of introgression between species, or the sharing of ancestral polymorphisms. Because the shared polymorphisms are present in almost all species pairwise comparisons in the case of *FBN9*, we argue that the effect of shared ancestral polymorphisms has played a key role in this pattern. In some cases the shared polymorphisms are as many as eight (*An. arabiensis*-*An. bwambae*) or 19 (*An. arabiensis*-*An. gambiae*).

Characterization of the FBN gene family has suggested that the FBNs have structural features (pattern recognition receptors) that allow them to recognize parasites and play an essential role in the mosquito's innate immunity, in addition to the physiological processes associated with blood feeding [28]. *FBN9 *a member of the FBN family, was recently found to be up-regulated when *An. gambiae *was invaded by *P. falciparum *ookinetes but not responding to *P. berghei *ookinetes invasion [11]. The structural features of FBN9, as well as the fact that in RNAi gene silencing assays, a *FBN9 *knockdown increases *An. gambiae *mosquitoes susceptibility to both *P. falciparum *and *P. berghei *infections [11], raise the possibility that this gene could be evolving in response to *Plasmodium*. Furthermore, in a recent study [29] it was shown that positive selection drives the evolution of pattern recognition receptors in *Drosophila*. However, even in the case of *FBN9 *no signs of positive selection could be detected and there were not even any fixed non-synonymous differences between the species (Table 4). *FBN9 *is also involved in the immune response against bacteria [11] and this could be the major reason why this gene as well has not undergone major adaptations to malaria.

Presently there are only three studies assessing genetic variation in innate immunity genes specifically implicated as being important in controlling *Plasmodium *infection in *Anopheles *[20,30,31]. In a study of the defensin gene [31]* An. gambiae*, *An. arabiensis *and *An. quadriannulatus *were involved and the authors concluded that strong purifying selection is acting on the mature peptide and probably the whole coding region. Furthermore, the authors argued that since *An. quadriannulatus *is not exposed to human pathogens, identical mature peptide and similar pattern of polymorphism across the three species implies that human pathogens played no role as selective agents on this peptide. Similarly, it was concluded [30] that no evidence for strong selection could be detected on a suite of mosquito immune system genes, *CTL4*, *CTLMA2*, *LRIM1*, and *APL2 *(or *LRRD7*), which have been shown to affect *Plasmodium *development in functional studies. The authors used five different species of the *An. gambiae *complex, namely *An. gambiae*, *An. arabiensis*, *An. bwambae *and *An. merus*. However, they only focused on *An. gambiae *and used all remaining species as outgroups in their analyses. One of the loci studied in [30], *LRIM1*, has been the subject of a separate study [20] in which besides the conservative McDonald-Kreitman tests for positive selection, a maximum likelihood approach with PAML software was also applied. In the latter study, six species of the *An. gambiae *complex were involved and it was concluded that *LRIM1 *underwent adaptive evolution in the *An. arabiensis *lineage.

*LRIM1 *was recently established as a major anti-*Plasmodium *factor [15] and it has been speculated that it could be possible that only some *LRIM1 *alleles suppress infection with *P. falciparum*, and these may even be specific for certain *P*.*falciparum *strains [20]. *LRIM1 *is the only gene of the *Anopheles *immune system that directly interacts with *Plasmodium *and has not been implicated to be involved in the defense against other pathogens. *LRIM1 *is also the only *Anopheles *immune gene that has been studied that shows strong signs of positive selection acting in one of the major malaria vectors, *An. arabiensis *[20].

## Conclusion

It is not surprising that studies of the genes considered here as well as those by [31] and [30] could not detect selection related specifically to *Plasmodium*. However, the positive results of [20] lend credence to the general comparative approach in identifying the minority of the hundreds of genes implicated in the insect innate immunity response that may have responded specifically to *Plasmodium*. Considering the results presented here in concert with previous studies, genes that interact directly with the *Plasmodium *parasite, and play little or no role in defense against other microbes, are probably the most likely candidates for a specific adaptive response against *P. falciparum*. However, even though several lines of evidence exist and support that that the *Anopheles *species have exhibited an adaptive response to *P*.* falciparum *infection [20], we cannot completely exclude the possibility that the mosquitoes are utilizing solely their anti-bacterial defense system to fight against *Plasmodium*. Furthermore, since it is hard to establish direct evidence linking the adaptation of any candidate gene to *P. falciparum *infection, a comparative framework allowing at least an indirect link should be provided. Such a framework could be achieved, if a similar approach like the one involved here, was applied to all other anopheline complexes that transmit *P. falciparum *malaria. Such complexes do exist (i.e. *Anopheles funestus, Anopheles nili, Anopheles moucheti*) in continental sub-Saharan Africa. Considering the systematic status of these anopheline complexes [32,33], it can be safely argued that the acquisition of *P. falciparum *by each of these complex of species was an independent evolutionary event. In the case that the same candidate gene and/or the same fragment of the respective gene, was identified as being evolving under positive selection in more than one of these complexes, then an indirect yet very strong proof of evolution imposed by *P. falciparum *infection, would be recognized.

## Methods

### Mosquitoes sampling

Six species of the *An. gambiae *species complex were included in this study. The adult *An. gambiae *specimens used were collected from two regions of Cameroon (Mbebé and Nyabéssan). Adult *An. arabiensis *females were collected from Kousseri in Cameroon, whereas adult *An. melas *were collected from Ipono (Cameroon). Larvae of *An. bwambae *originating from Bwamba county in Uganda, were kindly provided by Ralph Harbach. DNA extracts of *An. merus *were kindly provided by David O' Brochta (collected from Furvela in Mozambique). Finally, *An. quadriannulatus A *specimens from Kruger National Park in South Africa, were kindly provided by Anton Cornel.

### DNA methods

DNA was extracted using the DNeasy tissue kit (Qiagen) using either the entire mosquito or 2–3 of its legs. Species and molecular form identification was performed following recent diagnostic protocols [34,35]. All *An. gambiae *specimens belonged to the S molecular form.

### Loci analysed

We amplified four *An. gambiae *loci that have been experimentally associated with malaria infection. Multiple primers were designed for each one of the targeted loci using the software FastPCR [36] and based on the *An. gambiae *genome [3]. The amplified genes were: a) *Gambicin *(Ensembl Gene Id: AGAP008645, Chromosome 3R) that is composed of three exons and has a total length of 648 bp. Of these only 246 bp are coding sequence. Gambicin is an antimicrobial peptide that is ultimately secreted as a 61-aa mature peptide. It is induced during both early and late stages of malaria infection. In vitro experiments showed [19] that the mature peptide can kill both gram-positive and gram-negative bacteria, has a morphogenic effect on a filamentous fungus, and is marginally lethal to *Plasmodium berghei *ookinetes. The primer pairs designed in this study targeted the whole coding region of the gene and the introns in between. b) Nitric Oxide Synthase (*NOS*) [Ensembl Gene Id: AGAP008255, Chromosome 3R] which is a complex gene, and is comprised of 18 exons separated by introns of varying length and spread over about 33 kb (exons: 3342 bp). It was recently suggested [18] that *P. falciparum *ingestion triggers a midgut-associated, as well as a systemic, response in the mosquito, involving three genes, one of which is *NOS*. We targeted a fragment of approximately 1000 bp (part of exon 14, exons 15 and 16, part of exon 17 and all the introns between those exons). c) *Rel2 *(Ensembl Gene Id: AGAP006747, Chromosome 2L) that is comprised of 10 exons separated by introns of varying size. The total length of the gene is approximately 11.66 kb out of which only 3779 bp belong to exons. It has been found that this gene is involved [17] in the regulation of the intensity of mosquito infection with the malaria parasite, *P. berghei*. The primers used amplified a fragment of approximately 800 bp, including parts of exons 3 and 4 and the intron in between. d) *FBN9 *(Ensembl Gene Id: AGAP011197, Chromosome 3L) that is a single exon gene (846 bp) and produces a 282-aa mature peptide. *FBN9 *has been found to be up-regulated in *An. gambiae *after *P. falciparum *infection, but not with *P. berghei *infection [11]. The primers designed in this study aimed for a fragment of 810 bp covering almost the complete gene.

In most of the cases a nested PCR protocol was applied to successfully amplify the targeted loci. In the nested PCR protocol, the product of a PCR using a specific set of primers was used as a template for a subsequent PCR using primers internal to the ones used in the preceding PCR. The sequences of the primers used in the amplification of each locus are reported in Table 1. PCR products were examined on a 2% agarose gel, purified using the Qiaquick Purification Kit (Qiagen) and submitted for direct sequencing. The PCR products were sequenced in both directions using BigDye™ Terminator Cycle Sequencing Kit (v3.1, Applied BioSystems) reagents and an 3730 ABI capillary sequencer. All individuals that were found to be heterozygous for two or more positions were subjected to PCR amplification again and the amplicons were cloned using the TOPO-TA cloning kit for sequencing (Invitrogen). From each individual, a minimum of three transformed colonies were selected, and the size of the DNA insert was screened by PCR using the T3/T7 primer pair of the TOPO-TA vector. In most of the cases the correct size insert was obtained, and was subsequently sequenced in both directions. Because of multiple insertion/deletion (indels) in the introns of the *gambicin *locus, direct sequencing usually produced sequences of low quality. In order to circumvent this issue, most of the *gambicin *sequences produced were obtained via cloning of the PCR products. In this case, a minimum of five individually transformed colonies from each individual were screened, and at least three were sequenced. In all PCRs to ensure the minimum number of miss-incorporations Platinum High Fidelity Taq (Invitrogen) was used.

All produced sequence chromatograms were inspected by eye to confirm the validity of all differences either between alleles of the same individual, or within and between species. Sequences were viewed, edited and assembled using CodonCode Aligner (v. 1.6.3 CodonCode Corporation, Dedham, MA, USA). All produced sequences were compared to the published *An. gambiae *genome [3] to verify their homology to the respective loci. Sequences produced for this study have been submitted to GenBank under the accession numbers EU304549 to EU304787.

### Species polymorphism and divergence

All sequences were aligned using CodonCode Aligner (v. 1.6.3 CodonCode Corporation, Dedham, MA, USA). Basic analyses of polymorphism and divergence were performed using the computer program DNAsp v.4.10.3 [37]. Parameters estimated included the pairwise diversity (Pi) at synonymous and non-synonymous sites and the average number of nucleotide substitutions per site between species (Dxy).

### Tests for selection

In order to assess whether selection is acting on any of the immunity genes amplified from the six species of the *An. gambiae *complex, two different approaches were implemented. The first approach involved the McDonald-Kreitman test [38] that is intended to identify selection through an excess of amino acid substitution between species. This test compares the dN/dS ratio between species to within species and is based on the idea that substitutions under positive selection will go to fixation rapidly, and are therefore rarely observed as polymorphisms. However, they are present as fixed differences between species and an excess of replacement fixed differences is therefore an indication of positive selection. This test allows the detection of selection on a whole protein is bound to be quite conservative in detecting selection [39] and lacks the power of a site by site analysis. This analysis was performed using DNAsp v.4.10.3 [37].

A second and more powerful method to detect selection was also applied. This method detects elevated dN/dS ratios (ω ratios) using maximum likelihood approaches (see Yang 2007) and is less conservative than the McDonald-Kreitman test. We reasoned that it may be hard to detect positive selection on the whole of each amplified fragment, because the majority of their codons are likely to be functionally constrained and therefore under purifying selection. However, such purifying selection may be masking positive selection of a small number of codons within the amplified fragments. We therefore used a codon by codon maximum likelihood test, to ask if we could detect any codons that have been under repeated, strong positive selection. This method allows a site by site analysis, thus the identification of particular codons that have been evolving under selective constrains [40] is feasible. This analysis was performed using the software package PAML v. 4. [41]. At this point we have to stress that since we are dealing with a set of species very closely related, one has to be cautious in the interpretation of the results and the attribution of unusual patterns of diversity to selection. It is certain that shared ancestral polymorphisms and recent introgression between the complex members, will confound the actual processes. The loci subjected to the PAML analysis were those that exhibited at least one fixed non-synonymous change (i.e. *NOS *and *Rel2*), in some of the pairwise species comparisons (Tables 3, 4). Each locus was separately analyzed. Using sequence data from the coding and non-coding region of each amplified locus, a gene tree describing the phylogenetic relationships of all the taxa studied, was generated. The trees were constructed with the phylogenetic software program MrBayes 3.1 [42], using partitioned data. The data sets were partitioned so that a different substitution model could be applied to the introns, the first, second, and third codon positions of each gene. The substitution models implemented for each partition in the Bayesian analysis, were those suggested by Modeltest 3.7 [43] according to the Akaike Information Criterion [44]. The generated Bayesian trees served as the basis for the implementation of the maximum likelihood methods of the PAML package of programs [41] aiming at detecting adaptive molecular evolution under specific models of codon substitution. When sequences evolve under neutrality, the relative number of synonymous and non-synonymous substitutions is expected to be 1. In the case of positive selection, amino acid changes are favored and ω > 1, whereas under purifying selection amino acid changes are prevented and ω < 1.

In each one of the loci subjected to the PAML analysis, we estimated the likelihood values of the respective phylogenetic tree as being the result of lineages evolving under the assumptions of the site models M0, M3, M1a, M2a, M7 and M8 implemented in PAML. These models allow ω values to vary among different codons. Following the suggestions of Yang (2007) the site model pairs that appear to be particularly useful for real data analysis, are the M1a versus M2a and M7 versus M8. However, we also compared model M0 versus M3 in order to see if the selective pressure is uniform among sites. The strength of positive selection was calculated by comparing twice the log likelihood difference in a chi-square test with four (M0 versus M3) or two (M1a versus M2a, and M8 versus M7) degrees of freedom.

Finally, aiming at investigating whether the branches leading to the two major human malaria vectors, *An*.* gambiae *and *An*.* arabiensis*, are evolving under positive selection, we applied the branch-site models for each one of this species. Thus, the *An*.* gambiae *lineage in one case and the *An. arabiensis *lineage in the other case, were considered as the foreground branches (branch evolving with an ω value different than one), and ω values were allowed to vary among lineages and among sites [40]. Model 2, with ω free to vary (model = 2, several ω values for branches: settings of PAML), was compared to the same model, but with ω fixed to one (branch-site test 2) in order to examine if indeed the ω value of the foreground lineage is significantly different from one. The strength of positive selection was calculated by comparing twice the log likelihood difference in a chi-square test with 1 degree of freedom.

## Authors' contributions

AP produced the genetic data, carried out the analyses, and wrote the manuscript. JRP, and AC helped drafting the manuscript. JRP, AC and AP conceived and designed the experiments. JRP and AC coordinated the project. MAS, JCM, FS, PHA, CAN contributed reagents/materials and analysis tools. All authors read and approved the final manuscript.

## Supplementary Material

Additional file 1 ***Gambicin *Bayesian Inference Tree**. 50% majority-rule consensus Bayesian (unrooted) tree of *gambicin*. Numbers on branches are the posterior probabilities of clades, only values above 0.5 are presented. Species names have been abbreviated as follows: ARA: *An. arabiensis*, BWA: *An. bwambae*, GAM: *An. gambiae*, MEL: *An. melas*, MER: *An. merus*, and QUA: *An. quadriannulatus*. The number following the species abbreviation refers to the individual specimen code, whereas the letters A and B differentiate between the two alleles of a single individual specimen. Details of the Bayesian analysis can be provided upon request. Click here for file

Additional file 2 ***FBN9 *Bayesian Inference Tree**. 50% majority-rule consensus Bayesian (unrooted) tree of *FBN9*. Numbers on branches are the posterior probabilities of clades, only values above 0.5 are presented. Species names have been abbreviated as follows: ARA: *An. arabiensis*, BWA: *An. bwambae*, GAM: *An. gambiae*, MEL: *An. melas*, MER: *An. merus*, and QUA: *An. quadriannulatus*. The number following the species abbreviation refers to the individual specimen code, whereas the letters A and B differentiate between the two alleles of a single individual specimen. Details of the Bayesian analysis can be provided upon request. Click here for file
