# Supplementary material for: The molecular evolution of four anti-malarial immune genes in the Anopheles gambiae species complex
Source: BMC Evol Biol. 2008 Mar 6;8:79. doi: 10.1186/1471-2148-8-79 (PMC2288592; doi:10.1186/1471-2148-8-79)
Supplement: Supplementary file 1 [file 1471-2148-8-79-S1.doc]

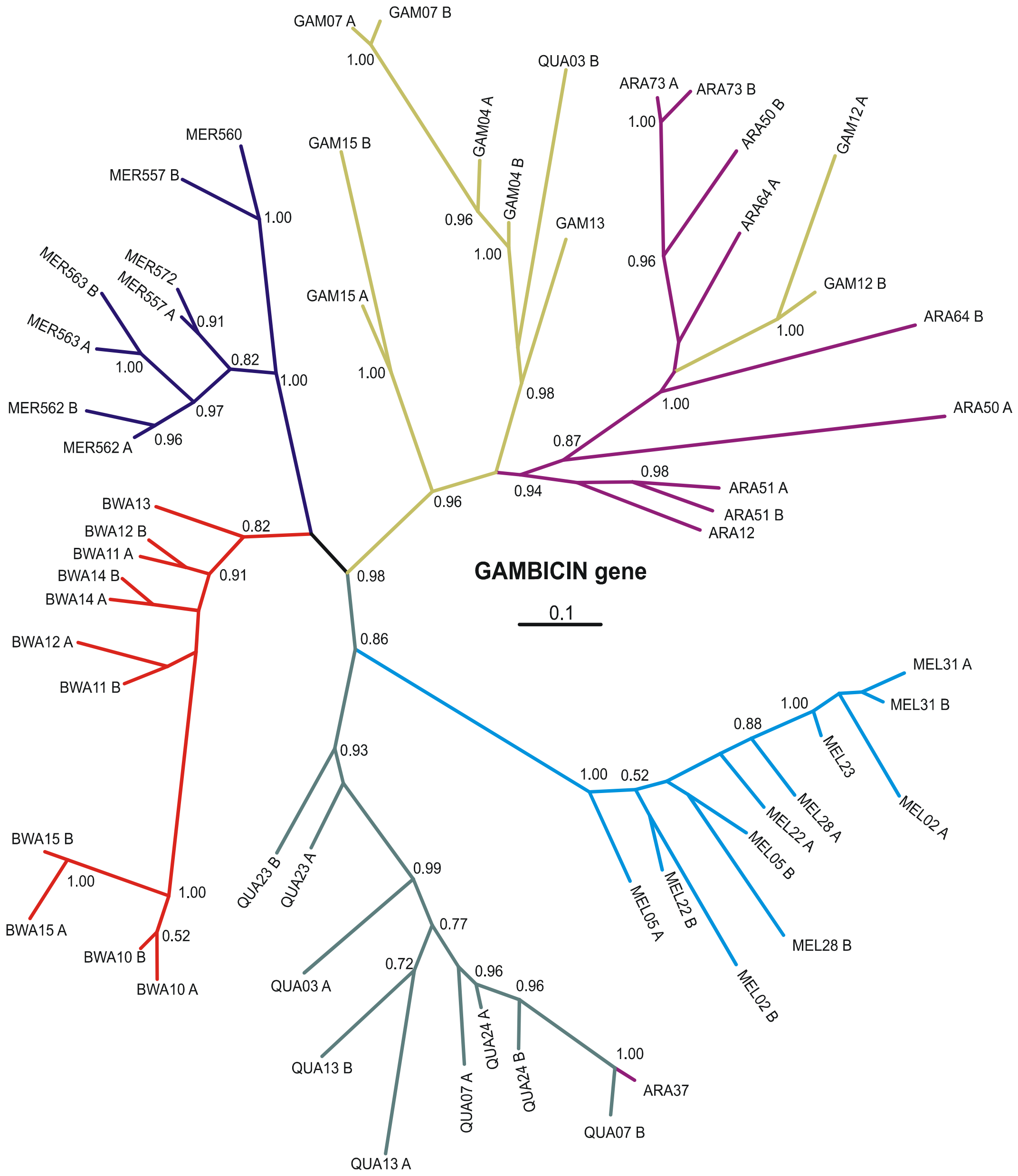
GAMBICIN gene
0.1
GAM07 A
GAM07 B
QUA03 B
ARA73 A
ARA73 B
GAM04 A
GAM04 B
GAM15 B
GAM13
GAM15 A
GAM12 A
GAM12 B
ARA50 B
ARA64 A
ARA64 B
ARA50 A
ARA51 A
ARA51 B
ARA12
MER560
MER557 B
MER572
MER557 A
MER563 B
MER563 A
MER562 B
MER562 A
BWA13
BWA12 B
BWA11 A
BWA14 B
BWA14 A
BWA12 A
BWA11 B
BWA15 B
BWA15 A
BWA10 B
BWA10 A
QUA23 B
QUA23 A
QUA03 A
QUA13 B
QUA13 A
QUA07 A
QUA24 A
QUA24 B
QUA07 B
ARA37
MEL31 A
MEL31 B
MEL23
MEL02 A
MEL28 A
MEL22 A
MEL05 B
MEL28 B
MEL02 B
MEL22 B
MEL05 A
