# Supplementary material for: The molecular evolution of four anti-malarial immune genes in the Anopheles gambiae species complex
Source: BMC Evol Biol. 2008 Mar 6;8:79. doi: 10.1186/1471-2148-8-79 (PMC2288592; doi:10.1186/1471-2148-8-79)
Supplement: Supplementary file 2 [file 1471-2148-8-79-S2.doc]

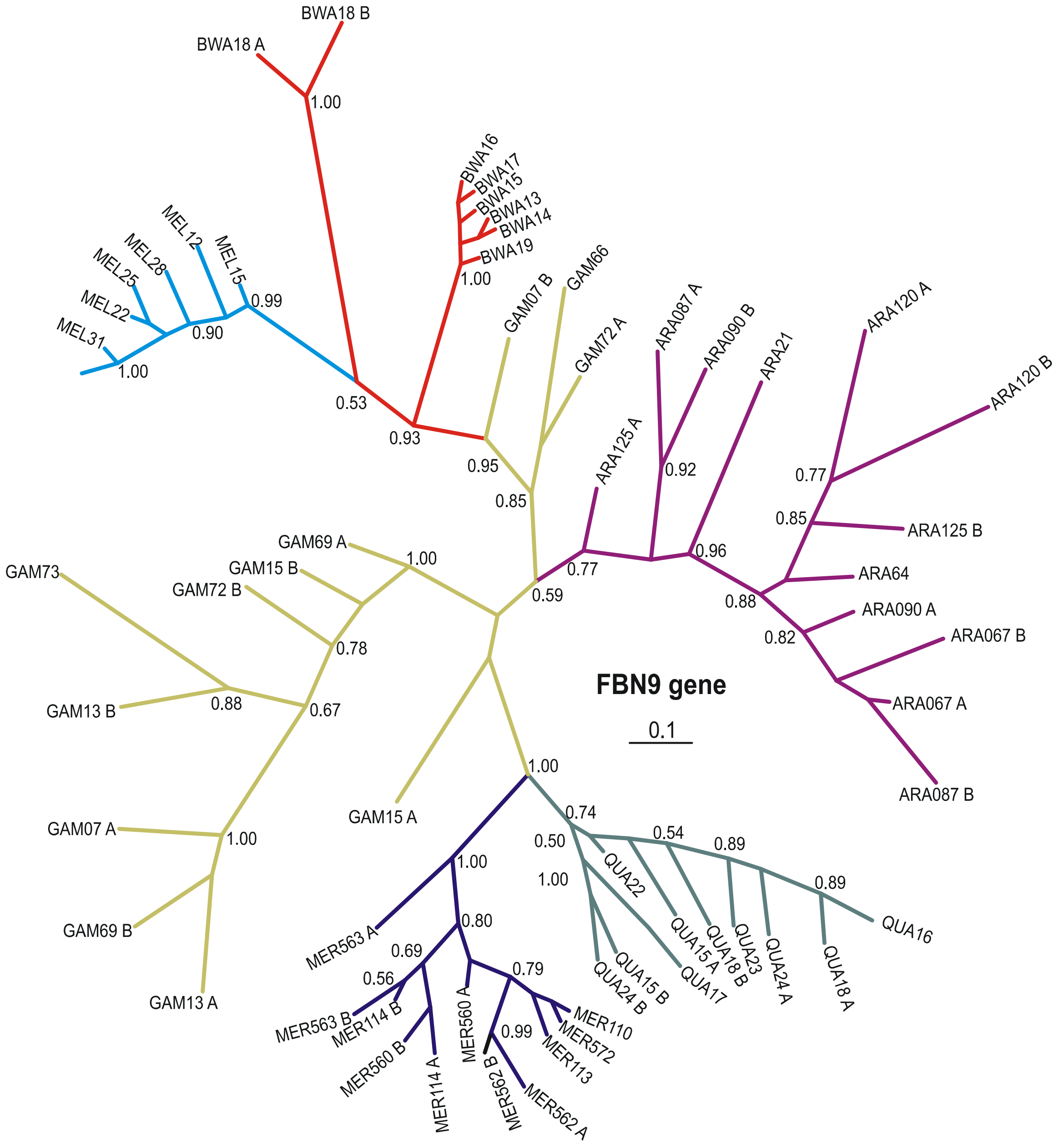
FBN9 gene
0.1
BWA18 A
BWA18 B
BWA16
BWA17
BWA15
BWA13
BWA14
BWA19
MEL12
MEL15
MEL28
MEL25
MEL22
MEL31
GAM07 B
GAM66
GAM72 A
GAM69 A
GAM15 B
GAM72 B
GAM73
GAM13 B
GAM07 A
GAM69 B
GAM13 A
GAM15 A
ARA087 A
ARA090 B
ARA21
ARA120 A
ARA120 B
ARA125 A
ARA125 B
ARA64
ARA090 A
ARA067 B
ARA067 A
ARA087 B
QUA22
QUA15 A
QUA18 B
QUA23
QUA24 A
QUA18 A
QUA16
QUA15 B
QUA24 B
QUA17
MER563 A
MER563 B
MER114 B
MER560 B
MER114 A
MER560 A
MER562 B
MER562 A
MER110
MER572
MER113
